# Protocol to investigate G protein-coupled receptor signaling kinetics and concentration-dependent responses using ONE-GO biosensors

**DOI:** 10.1016/j.xpro.2024.103383

**Published:** 2024-10-11

**Authors:** Remi Janicot, Mikel Garcia-Marcos

**Affiliations:** 1 Department of Biochemistry & Cell Biology, Chobanian & Avedisian School of Medicine, Boston University, Boston, MA 02118, USA; 2 Department of Biology, College of Arts & Sciences, Boston University, Boston, MA 02115, USA

**Keywords:** biophysics, cell biology, cell culture, cell-based assays, molecular biology, signal transduction, biotechnology and bioengineering

## Abstract

ONE vector G protein optical (ONE-GO) biosensors are versatile tools to measure the activity of G protein-coupled receptors (GPCRs) in cells. The availability of ONE-GO biosensors for ten active Gα subunits representative of all four G protein families (G_s_, G_i/o_, G_q/11_, and G_12/13_) permits the study of virtually any GPCR. Here, we present a protocol to implement ONE-GO biosensors in cell lines to investigate GPCR signaling kinetics and concentration-dependent responses. We describe steps for cell culture and transfection, response measurement, and data analysis.

For complete details on the use and execution of this protocol, please refer to Janicot et al.^1^

## Highlights

• Instructions for transfecting HEK293T cells for BRET-based GPCR activity assays
• Protocol for rapid kinetic measurements of GPCR activation with ONE-GO sensors
• Guidance for scalable endpoint assays to measure concentration-dependent responses

## Abstract (editor highlights)

Publisher’s note: Undertaking any experimental protocol requires adherence to local institutional guidelines for laboratory safety and ethics.

## Graphical abstract

**Figure:**
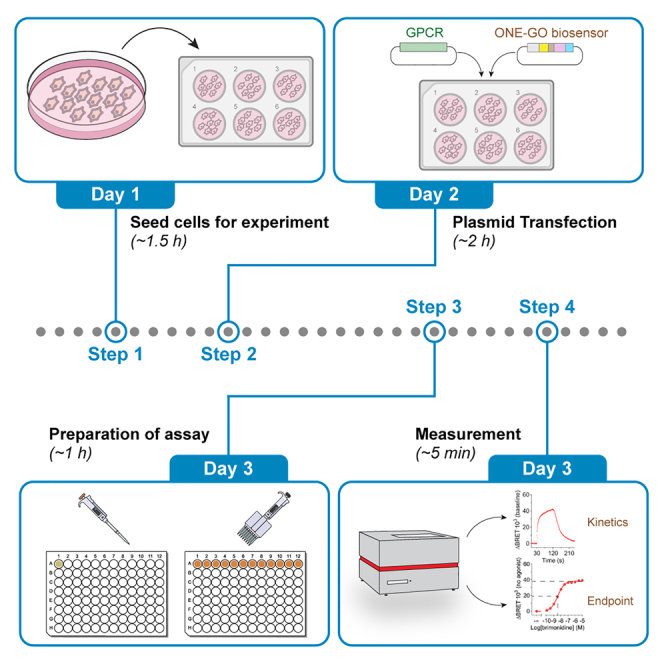

## Before you begin

### Background

G protein-coupled receptors (GPCRs) are a large family of evolutionarily conserved transmembrane proteins that respond to a remarkable variety of extracellular stimuli to evoke cellular responses. In humans, GPCRs trigger responses elicited by every major neurotransmitter, the majority of hormones, countless odorants, or even photons, among other stimuli.^2^^,^^3^^,^^4^^,^^5^ Consistent with the broad range of physiological functions controlled by GPCRs, their dysregulation is also linked to a variety of diseases and disorders. GPCRs stand out as pharmacological targets, given that they represent the target for over one-third of drugs used in the clinic and the continued interest in targeting them through many ongoing campaigns and clinical trials in varying stages of development.^6^^,^^7^ GPCRs primarily signal through heterotrimeric G proteins (Gαβγ), although arrestins have also been shown to mediate some responses.^3^^,^^4^ Active GPCRs function as guanine nucleotide exchange factors (GEFs) for heterotrimeric G proteins, provoking conformational changes within the Gα subunit that promote the exchange of GDP for GTP, and a subsequent dissociation of Gβγ. Both Gα-GTP and “free” Gβγ are the active species that propagate signaling inside the cell by acting on downstream effectors to evoke specific cellular responses.^8^^,^^9^

Given the prevalence and importance of processes initiated by GPCRs and G proteins, precise and accurate measurement of the signal transduction events they mediate is crucial for advancing the understanding of biological processes and the mechanisms of action of many drugs. Traditional methods to monitor GPCR-mediated G protein activation include measuring downstream readouts like second messengers (e.g., cAMP, intracellular Ca^2+^) or gene reporters,^10^^,^^11^ but these methods are affected by signal amplification and/or crosstalk events that skew results. A general approach to mitigate these potential limitations is to leverage biosensors that directly detect the activation status of G proteins — i.e., measuring dissociation of Gαβγ heterotrimers or GTP loading on Gα.^12^^,^^13^^,^^14^^,^^15^^,^^16^^,^^17^^,^^18^^,^^19^

**Figure 1:**
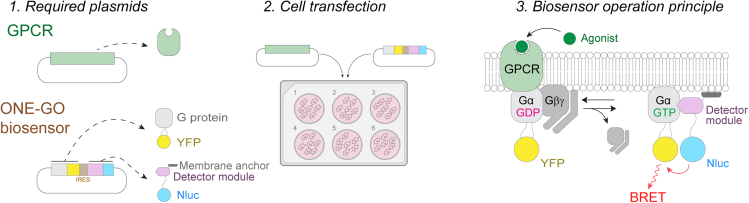
Overview of ONE-GO biosensor design principle and its application in cell lines The only required expression plasmids are a GPCR of interest and a single ONE-GO biosensor plasmids, which will lead to the expression of both a YFP-tagged Gα subunit and an Nluc-tagged detector module. After GPCR stimulation, the YFP-tagged Gα subunit adopts an active conformation that specifically binds to an Nluc-tagged detector module, resulting in BRET.

While there have been many G protein biosensor designs generated over the last decades, a recently developed platform named ONE-Vector G protein Optical (ONE-GO) biosensors offers some unique advantages. These biosensors, which are based on bioluminescence resonance energy transfer (BRET), operate with virtually any GPCR, can be implemented in a wide variety of cell types, do not interfere with the normal function of the signaling pathways under investigation, and are easy to implement based on their “ready-to-use” design as a single plasmid.^1^ Essentially, ONE-GO biosensors consist of a “detector module” that specifically binds to GTP-bound Gα subunits fused to a BRET donor (Nluc) and a Gα tagged with a BRET acceptor (YFP) at a safe internal location of the protein. The detector module fused to Nluc is constitutively attached to membranes through lipidic anchor and recognizes specifically the active form of Gα by directly binding to a region of the G protein that changes conformation upon GTP loading. Thus, when a GPCR promotes the activation of the YFP-fused Gα in cells, binding of the detector module to the G protein brings the fused BRET donor Nluc in close proximity to the acceptor, resulting in an increase of BRET. In the ONE-GO biosensor design, both the Gα-YFP and the Nluc-fused detector module are expressed simultaneously from a single plasmid in ratios appropriate to detect robust responses, while maintaining low expression levels of the exogenous components to minimize interference with endogenous signaling^1^ (Figure 1). Because higher acceptor-to-donor ratios are conducive to observing larger differences in BRET, the donor-containing component of ONE-GO biosensors is expressed downstream of a low efficacy IRES sequence,^20^^,^^21^ such that its expression is lower than that of the acceptor-containing component of the biosensor located right downstream of the promoter driving the expression of the whole cassette.

Here, we provide detailed protocols for using ONE-GO biosensors to monitor G protein activity with subsecond resolution in a kinetic assay, or to generate concentration-dependent curves for GPCR ligands in an endpoint assay.

***Note:*** This protocol only illustrates how to use the Gαi1 ONE-GO sensor to detect α2_A_-adrenergic receptor (α2_A_-AR) responses, but the same instructions can be followed for other combinations of Gα ONE-GO biosensor and GPCR.

***Note:*** While ONE-GO biosensors can also be implemented to measure responses triggered by endogenous GPCRs (instead of those triggered by exogenous GPCR as described in this protocol), the ability to detect such responses depends on whether the receptor of interest is expressed in the cell type of interest. The companion manuscript with title “*Detecting endogenous GPCR activity in primary cell cultures using ONE-GO biosensors*” provides an example protocol for the implementation of ONE-GO biosensors to detect endogenous GPCR responses.

***Note:*** ONE-GO biosensors are also compatible with lentiviral packaging and transduction into cell types that are not easily transfected (e.g. primary cells). A protocol for this use of ONE-GO biosensors in primary cells is described in the companion manuscript with title “*Detecting endogenous GPCR activity in primary cell cultures using ONE-GO biosensors*”.

## Key resources table

**Table:** 

REAGENT or RESOURCE	SOURCE	IDENTIFIER
**Chemicals, peptides, and recombinant proteins**		

Brimonidine	Thermo Fisher Scientific	Cat #461520010
Yohimbine	Alfa Aesar	Cat #J60185
Fetal calf serum (FCS)	Cytiva	Cat #SH30073.03
100x penicillin-streptomycin-L-glutamine (PSG)	Corning	Cat #30-009-Cl
DMEM, high glucose	Gibco	Cat #11965-092
Trypsin, 0.25% (w/v)-2.21 mM EDTA	Corning	Cat #25-053-Cl
Hank’s balanced salt solution (HBSS)	Corning	Cat #21-022-CV
Phosphate-buffered saline (PBS)	Corning	Cat #21-040-CV
Gelatin type-B, 2% (w/v)	Sigma-Aldrich	Cat #G1393
NaCl	Fisher Scientific	Cat #BP358-10
HEPES	Fisher Scientific	Cat #BP310-500
NaH_2_PO_4_	Fisher Scientific	Cat #BP329-500
CaCl_2_·2H_2_O	Fisher Scientific	Cat #BP510-100
KCl	Fisher Scientific	Cat #P333-500
MgCl_2_·6H_2_O	Fisher Scientific	Cat #BP214-500
Glucose	Fisher Scientific	Cat #D16-500

**Critical commercial assays**		

Nano-Glo Luciferase Assay System	Promega	Cat #N1120
CTZ400a	GoldBio	Cat #C-320
Coelenterazine	Promega	Cat #S2001

**Deposited data**		

ONE-GO Biosensors Kit and related plasmids (including full plasmid sequences)	Addgene	Kit #1000000224

**Experimental models: Cell lines**		

HEK293T	ATCC	Cat #CRL-3216

**Recombinant DNA**		

pLVX-CMV-Gαi1-YFP-IRES∗-KB1753-Nluc (Gαi1 ONE-GO)	Janicot et al.^1^	N/A
pcDNA3-α2_A/D_-AR (rat)	Oner et al.^22^; Oner et al.^23^	N/A

**Software and algorithms**		

GraphPad Prism	GraphPad Software	https://www.graphpad.com/scientific-software/prism/
Adobe Illustrator	Adobe	https://www.adobe.com/

**Other**		

POLARstar Omega (microplate reader)	BMG Labtech	N/A
10-cm tissue culture plate	Thermo Scientific	Cat #130182
6-well tissue culture plate	Corning	Cat #3516
Polystyrene transfection tubes	Fisher Scientific	Cat #FB149563A
96-well plate	Thermo Scientific	Cat #268152
White 96-well assay plate	PerkinElmer	Cat #6005290
Cell scraper	Thermo Scientific	Cat #179693
*(Optional: Electronic multi-channel pipet)*	*Sartorius*	*Cat #LH-747361*

## Materials and equipment

**Table:** HEK293T DMEM

Reagent	Final concentration	Amount
FCS	10%	50 mL
PSG (100×)	1×	5 mL
DMEM, high glucose	N/A	Up to volume
**Total**	**N/A**	**500 mL**

Store at 4°C for up to 4 weeks.

Culture media can be substituted if established protocols are already in place in other laboratories.

### Trypsin-EDTA, 0.05% (w/v)

• Dilute 100 mL of trypsin, 0.25% (w/v)-2.21 mM EDTA in HBSS for a final volume of 500 mL.

***Note:*** Make 50 mL aliquots before storing. [Store at −20°C for up to 6 months; keep “in-use” aliquot at 4°C for up to 8 weeks]. Other reagents for cell detachment can be used as long as they are established and validated for the cell type of interest.

### Gelatin, 0.1% (w/v)

• Dilute 1 mL of gelatin type-B, 2% (w/v) in ddH_2_O for a final volume of 20 mL.

[Store at 4°C for up to 2 weeks].

Coating of cell culture wells can be substituted with other commonly used reagents to increased cell adherence like poly-L-lysine (Millipore Sigma, cat# P9155).

**Table:** 2× HEPES-Buffed Saline (HBS) solution

Reagent	Final concentration	Amount
NaCl	280 mM	8.18 g
HEPES	50 mM	5.96 g
NaH_2_PO_4_	1.5 mM	90 mg
ddH_2_O	N/A	Up to volume
**Total**	**N/A**	**500 mL**

**CRITICAL:** Precisely adjust pH to 7.4, then filter sterilize by passing through a 0.45-μm filter

***Note:*** Make 15 mL aliquots before storing. [Store at −20°C for up to 8 months; keep “in-use” aliquot at 4°C for up to 8 weeks]. Other transfection methods (e.g., lipofection) can be used in place of calcium-phosphate.

### 2 M CaCl_2_

• Dissolve 147.02 g of CaCl_2_·2H_2_O powder in ddH_2_O filled up to 500 mL to reach 2 M concentration.

***Note:*** Filter sterilize by passing through a 0.45-μm filter and make 15 mL aliquots before storing. [Store at −20°C for up to 8 months; keep “in-use” aliquot at 4°C for up to 8 weeks]. Other transfection methods (e.g., lipofection) can be used in place of calcium-phosphate.

**Table:** BRET Buffer

Reagent	Final concentration	Amount
NaCl	140 mM	8.18 g
KCl	5 mM	0.373 g
MgCl_2_	1 mM	0.203 g
CaCl_2_	1 mM	0.147 g
NaH_2_PO_4_	0.37 mM	0.044 g
HEPES	20 mM	4.76 g
Glucose	0.1%	1 g
ddH_2_O	N/A	Up to volume
**Total**	**N/A**	**1 L**

**CRITICAL:** Adjust pH to 7.4 before binding to the final volume and then filter sterilize by passing through a 0.45-μm filter.

***Note:*** Make 50 mL aliquots before storing. [Store at −20°C for up to 12 months; keep “in-use” aliquot at 4°C for up to 6 weeks]. Other physiologic saline, isotonic buffers can be used as long as they preserve cell viability and do not contain components that interfere with the luminescent emissions (e.g., color dyes like phenol red).

### 100 × 10 μM CTZ400a

• Dissolve 2 mg CTZ400a in 5.12 mL 200-proof EtOH.

**CRITICAL:** Keep away from light as much as possible.

***Note:*** Aliquot in screw-cap 1.5 mL tubes. [Store at −20°C for up to 12 months]. Other luciferase substrates for nanoluciferase, like Nano-Glo (Promega, cat# N1120) or Coelenterazine (Promega, cat# S2001), may be used for this assay.

## Step-by-step method details

### Culture of HEK293T cells and seeding for experiments

**Timing: 1.5 h if starting from confluent plate of cells**

**Figure 2:**
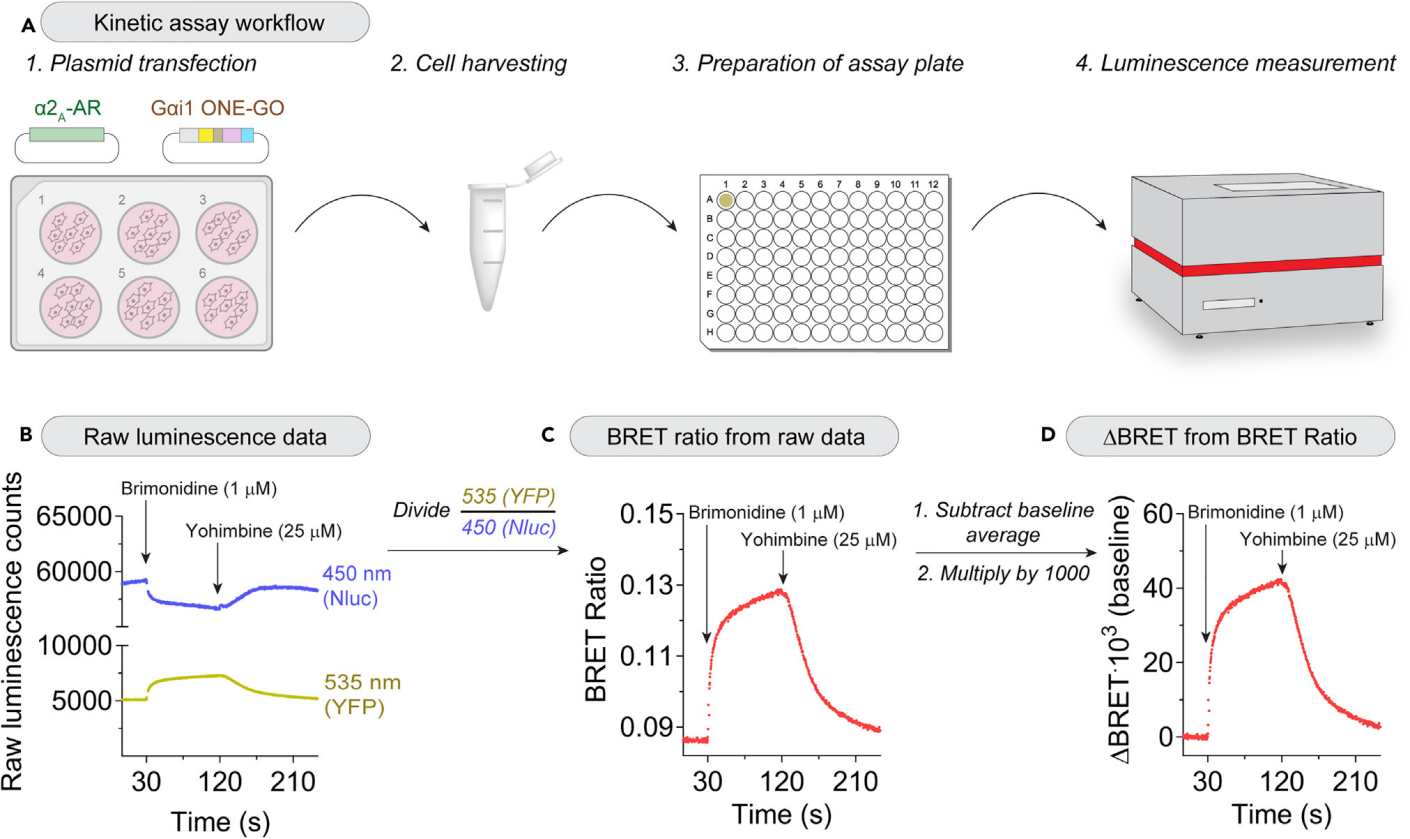
Kinetic measurements of Gα-GTP with the Gαi1 ONE-GO biosensor in HEK293T cells upon α2_A_-AR modulation (A) Schematic representation of experimental protocol. (B) Time traces for the simultaneous detection of luminescence at both 450 nm channel (Nluc, BRET donor) and 535 nm channel (YFP, BRET acceptor). (C) BRET ratio signals upon stimulation of the α2_A_-AR with brimonidine and inhibition with yohimbine, representing changes in cellular levels of Gαi1-GTP. (D) Processing of data in (C) to represent changes in BRET (ΔBRET). The BRET ratio values during the 30 s prior to agonist injection were averaged and subtracted from all data points and the resulting values multiplied by 1000 to represent results with more intuitive numbers.

This section describes how to handle and culture HEK293T cells, and how to seed them prior to a transfection and subsequent activity assay (Figure 2A).

1. Have a stock 10-cm plate of HEK293T cells cultured in HEK293T DMEM ready for experiments.
  a. Cells should be ∼90% confluent at the time of seeding.

**CRITICAL:** Never let cells grow over 95 % confluency as it will affect cell health. See troubleshooting 1.

2. Coat wells of a 6-well plate with 0.1% gelatin solution.
  a. Add 1 mL of gelatin, 0.1% to each well.
  b. Tilt plate to spread gelatin all over the well, then let the plate sit for 5 min.
  c. Aspirate gelatin and leave plate without a cover to air-dry.
3. Trypsinize HEK293T cells and harvest.
  a. Wash stock plate of cells with 5 mL of PBS.
    i. Add PBS slowly against the wall of the plate to avoid detaching cells.
  b. Aspirate PBS, and add 1 mL of trypsin-EDTA, 0.05% (w/v).
    i. Tilt 10-cm plate to spread the trypsin-EDTA, and place in 37°C incubator for ∼1 min.
  c. Confirm that the majority of cells are detached by visually inspecting the plates under a microscope.
  d. Stop trypsinization after by adding 5 mL of HEK293T DMEM.
  e. Transfer cell suspension to a 50 mL conical tube and dilute up to 20 mL with HEK293T DMEM.

***Note:*** This dilution ratio allows for a cell confluency that is neither too high nor too low to allow for cell counting and seeding. It can be adjusted as needed.

4. Count cells using e.g., a hemocytometer.

**CRITICAL:** Before pipetting the aliquot of cells to count, make sure you mix the cells well to have a homogenous suspension.

5. Seed 350,000 cells per well in a final volume of 2 mL media in a 6-well plate.
6. Place 6-well plate back in incubator and wait until following morning to perform the transfection.

***Note:*** One well of a 6-well plate should provide enough cells for both the kinetic and end-point assays described below.

***Note:*** The amount of cells seeded/transfected can be adjusted based on experimental needs. Each well seeded will correspond to one transfection condition. Potential control wells could include untransfected cells, cells transfected with biosensor but no exogenous GPCR, and an extra well with cells transfected with both biosensor and exogenous GPCR that is left unstimulated for comparison with other equivalent wells undergoing stimulation.

### Plasmid transfection

**Timing: 2 h**

This section describes how to perform a calcium-phosphate transfection to introduce the necessary components (i.e., a GPCR and a ONE-GO biosensor) into HEK293T cells.

***Note:*** Other methods of transfection (e.g. polyethylenimine [PEI] or lipofectamine) are also compatible with ONE-GO sensors but will not be covered here.

7. Warm up HEK293T DMEM, 2x HBS, 2 M CaCl_2_, and ddH_2_O in a 37°C water bath.
8. Change media of seeded cells.
  a. Aspirate media and replace with 1.5 mL of fresh HEK293T DMEM per well.
  b. Place 6-well plate back in the incubator.

***Note:*** At this stage, cells should be 50%–60% confluent in each well of a 6-well plate.

***Note:*** Add new media slowly against the wall to avoid detaching cells.

9. Add 122 μL 2x HBS in a 5-mL polystyrene tube.
10. In another 5-mL polystyrene tube, mix 105.6 μL ddH_2_O and 14.4 μL 2 M CaCl_2_.
11. Add DNA to the tube with CaCl_2_.
  a. Add the following amounts of plasmid DNA: 200 ng α2_A_-AR; 50 ng Gαi1 ONE-GO sensor. See troubleshooting 2, troubleshooting 3, or troubleshooting 4.

**CRITICAL:** The total volume of plasmids added should be kept under 10 μL to avoid affecting the mixing and transfection efficiency.

**CRITICAL:** The amount of biosensor expressed is crucial for the success of the experiment. Initially, we recommend doing a titration to find the best condition (e.g. transfect 10 ng, 50 ng, 100 ng, or 200 ng of the ONE-GO biosensor plasmid and compare side-by-side). While 200 ng of GPCR transfected should work well for most receptors, titrating down the amount of receptor transfected can sometimes facilitate detecting responses, especially in cases of receptors with high constitutive activity that may mask agonist induced responses.

**CRITICAL:** Total luminescence counts do not necessarily correlate with the larger BRET responses. In other words, expressing more of the biosensor to get higher counts is not always a good strategy to detect a better BRET response, and in many cases tends to lead to worse responses. See troubleshooting 3.

12. Vortex tube with DNA at medium speed for 5 s and let it sit for ∼5 min.
13. Add the entire volume of the tube with CaCl_2_ and the DNA to the tube with 2x HBS.

**CRITICAL:** Performing this step accurately is important to form the fine precipitate required for efficient transfection. Add volume slowly and dropwise while vortexing. For this, set the vortex at a moderate speed (e.g., ∼30% of the maximum speed) and keep it on (not in “press mode”). Hold the 2x HBS tube on it with one hand, so it shakes in place in a controlled manner that allows pipetting the contents of the CaCl_2_ solution with the other hand. See troubleshooting 1.

14. Let tubes sit at 18°C–23°C for 25 min.
15. Add entire volume of transfection mix dropwise to cells and mix by gently rocking plate.

***Note:*** If there is a need for replicate wells of a particular transfection condition, a larger transfection master mix can be made in a larger tube (e.g., for triplicate conditions, use 366 μL 2x HBS [122 μL × 3], 316.8 μL ddH_2_O [105.6 μL × 3], 43.2 μL 2M CaCl_2_ [14.4 μL × 3], and scale up 3-fold the amounts of plasmid transfected to be mixed in a 15 mL conical tube) and split between wells. In this case, it is required to gently pipet after the 25 min incubation to resuspend the precipitate before adding to cells.

16. Place cells back in incubator, and replace media ∼6 h later with 2 mL of HEK293T DMEM preheated in a 37°C water bath.
17. The following day, perform luminescence measurements.

***Note:*** Some GPCRs can get desensitized in HEK29T DMEM and may require ∼18 h starvation before the assay. See troubleshooting 4.

### Kinetic assay of G protein activity

**Timing: 1 h**

This section describes how to harvest cells for experiment and perform BRET measurements to directly monitor G protein activation or deactivation with sub-second resolution.

18. Warm up PBS and BRET buffer to 18°C–23°C.
19. Wash cells with PBS.
  a. Aspirate media, then add 2 mL PBS slowly against the wall.
  b. Aspirate PBS then add 1 mL of fresh PBS.
20. Scrape cells in the PBS and transfer them to 1.5 mL microcentrifuge tube.

***Note:*** Harvesting and measuring cells in suspension is tolerated well by HEK293 cell and provides flexibility in terms of adjusting the final number of cells per well, as well as other steps of the workflow. However, it is also possible to assay cells directly in a 96-well plate without harvesting in suspension, which would require setting up conditions of seeding and transfection precisely.

21. Centrifuge cells at 500 × *g* for 5 min at 18°C–23°C.
22. Aspirate supernatant and gently resuspend cells in ∼900 μL BRET buffer.

**CRITICAL:** Keep cells in dark after this step to avoid photobleaching of the biosensor components.

23. Prepare a 20 μM brimonidine and a 500 μM yohimbine solution diluted in BRET buffer.
  ***Note:*** Brimonidine and yohimbine are used as prototypical compounds to specifically activate or inhibit, respectively, α2-adrenergic receptors. If another GPCR is transfected, different agonists and antagonists should be used.
  ***Note:*** The concentrations indicated are 20x stock solutions that will be injected to the assay wells to achieve a concentration of 1 μM and 25 μM, respectively, upon injection of 5 μL into a final volume of 100 μL in the assay plate.
  **CRITICAL:** Avoid freeze-thaw cycles of stock chemicals, make aliquots as needed.
  a. Prime one injection pump in the plate reader with the 20x brimonidine (agonist) stock solution, and another pump with the 20x yohimbine (antagonist) stock solution.
24. In one well of a 96-well assay plate, add in the following order: 45 μL of BRET buffer, 50 μL of cells, 0.5 μL Nano-Glo.
  **CRITICAL:** Gently resuspend cells in the 1.5 mL microcentrifuge tube by pipetting before withdrawing aliquots to be added to the assay wells, as cells may settle to the bottom of the tube over time
  a. Gently mix the components in the assays well by pipetting after adding Nano-Glo.
25. Place plate in a plate reader equilibrated at 28°C.

***Note:*** Measurements can also be done a physiologic temperature (i.e., 37°C) with similar results but of somewhat reduced quality due to faster turnover of the luciferase substrate and modest reductions in BRET response amplitudes. The choice of 28°C is motivated by the desire to maintain temperature constant across all experiments regardless of laboratory environmental conditions. Typically, the lowest temperature inside the instrument is 3°C–4°C above room temperature, so setting it up to 28°C ensures consistency regardless of range of temperature typically observed in the laboratory.

26. Wait 2 min before starting measurement. See troubleshooting 2.

***Note:*** Setting an integration time of 0.24 s provides a good balance of time resolution and light detection sensitivity, but can be adjusted based on the intensity of the signal. See troubleshooting 1.

27. Measure simultaneously the emission centered at 450 nm with a bandpass of 80 nm (nanoluciferase luminescence) and emission centered at 535 nm with a bandpass of 30 nm (YFP emission).
  a. Inject 5 μL brimonidine 30 s after starting the measurement.
  b. Inject 5 μL yohimbine at 120 s, and stop measurement at 240 s. See troubleshooting 5.

***Note:*** Depending on the GPCR and/or G protein the activation and deactivation rates can differ. The times of agonist/antagonist injections and total measurement times can be adjusted as needed.

**CRITICAL:** It is desirable but not essential to use a plate reader that allows for simultaneous measurements of luminescence from both the donor and acceptor channel, which increases the technical reproducibility of the reads. Similarly, instruments with more sensitive detectors are also preferred to reduce noise. Plate readers that allow to record while injecting, as opposed to those that require to stop recording during injections, also allow for better kinetic measurements without loss of information.

28. To process the BRET data, export raw luminescence values from both the 535 nm (YFP) and 450 nm (Nluc) channels (Figure 2B).
  a. Divide raw luminescence counts in the acceptor channel (YFP) by raw luminescence counts in the donor channel (Nluc) for each time point (BRET ratio) (Figure 2C).
  b. Calculate the average BRET ratio for the 30 s preceding injection of the agonist to determine basal BRET signal.
  c. Subtract this average from all data points to get a difference in BRET relative to baseline (ΔBRET [baseline]) and multiply by 1000 (ΔBRET·10^3^ [baseline], Figure 2D) to obtain non-fractional numerical values that are easier to grasp and discuss.

***Note:*** At this point, there should be a 30 s baseline around the 0 value, followed by an increase in BRET post agonist injection which is reverted after antagonist injection (Figure 2D).

### End-point assay for concentration-dependent G protein activation

**Timing: 1 h**

**Figure 3:**
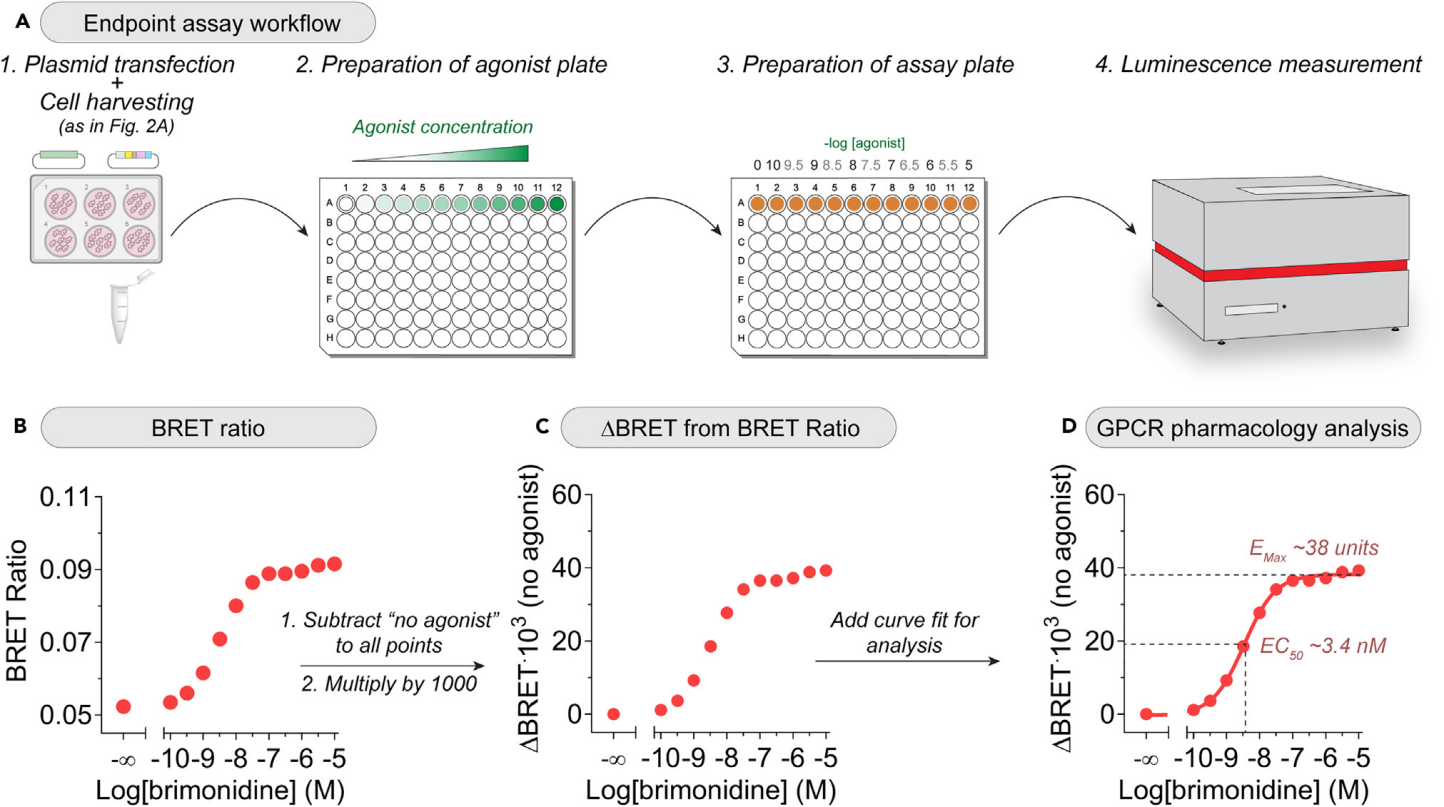
Endpoint measurements of Gα-GTP with the Gαi1 ONE-GO biosensor in HEK293T cells upon concentration-dependent stimulation of α2_A_-AR (A) Schematic representation of experimental protocol. (B) BRET ratio signals (ratio of acceptor/ donor luminescence signals) upon stimulation of the α2_A_-AR with different concentrations of brimonidine, representing changes in cellular levels of Gαi1-GTP. (C) Processing of data in (B) to represent changes in BRET (ΔBRET). The BRET ratio values in the conditions without brimonidine stimulation were subtracted from all data points and the resulting values multiplied by 1000 to represent results with more intuitive numbers. (D) Fitting of data from (C) to a 3-parameter sigmoidal curve to estimate the E_Max_ and the EC_50_.

This section describes how to perform BRET measurements to assess concentration-dependent GPCR responses and extract pharmacological parameters like efficacy (E_Max_) or the half maximal effective concentration (EC_50_) (Figure 3A).

29. Cell culture and transfection should be followed as explained for the kinetic assay (see steps 1–17).
30. To harvest cells for experiments, follow steps 18–22.
  a. Resuspend cells in ∼400 μL BRET buffer after centrifugation step (instead of resuspending cells in ∼900 μL BRET buffer as in step 22).
31. Prepare a 50 μM brimonidine solution (corresponding to 5 × 10 μM brimonidine, the highest concentration of agonist desired for the curve) in BRET buffer and add it to well #12 (rightmost well) of a 96-well round-bottom plate.

***Note:*** This 5x agonist dilution will be diluted to 1x during the preparation of the assay plate below.

**CRITICAL:** Reaching saturation of GPCR/G protein activation should be achieved by using a supramaximal dose of agonist, which will be shown as a plateau of BRET across the highest concentrations of agonist.

32. Dilute brimonidine in BRET buffer in 0.5 logs (which corresponds to a serial 3.16x dilution).
  a. For example, transfer 100 μL of a given concentration of agonist to the adjacent well on its left containing 216 μL BRET buffer, and repeat the process until reaching well #2 (second well form the left).

**CRITICAL:** Mix thoroughly at every dilution step to achieve accurate concentrations without propagation of an error during serial dilution.

33. Leave well #1 (leftmost well) with BRET buffer only (i.e., no agonist).

***Note:*** At this point you should have 12 different dilutions prepared in a row of a 96-well plate going from BRET buffer only in well #1 to a maximum dose of 50 μM brimonidine (i.e., 5 × 10 μM brimonidine) in well #12.

34. Prepare the assay plate by first transferring 20 μL of each diluted 5x agonist to a row of a 96-well assay plate (BRET buffer in well 1; highest dose in well 12).
35. Add 35 μL BRET buffer to each well of the assay plate.
36. Prepare a CTZ400a master mix by mixing 1 μL of 100x CTZ400a with 21.5 μL of BRET buffer/well.

***Note:*** The main reason to use CTZ400a instead of Nano-Glo is cost. While Nano-glo is somewhat better than CTZ400a in terms signal stability over time, scaling up for end-point measurements, which are less demanding than kinetic measurements in terms of signal stability over time, becomes more cost-effective with CTZ400a. Our experience is that results using either Nano-Glo or CTZ400a in this assay are undistinguishable from each other.

***Note:*** Account for some excess when preparing the CTZ400a master mix. For example, for 12 wells, prepare a master mix volume equivalent to the need for 15 wells— i.e., mixing 337.5 μL BRET buffer and 15 μL CTZ400a.

***Note:*** Other luciferase substrates compatible with the Nluc enzyme can be used, like Nano-Glo or coelenterazine.

37. Add 22.5 μL CTZ400a master mix to each assay well.

***Note:*** Adding the CTZ400a master mix using the multi-dispense function of an electronic pipet helps, but fast manual pipetting also works.

38. Add 22.5 μL of cell suspension prepared in step 30 to each well of the assay plate to start stimulation with the agonist already in the plate.

***Note:*** Gently mix the cell suspension before pipetting into the wells to ensure the addition of a consistent number of cells per well.

***Note:*** Adding the cells using the multi-dispense function of an electronic pipet helps to start stimulation at similar time points, but fast manual pipetting also works.

***Note:*** We have observed that optimal results are achieved when pipetting the CTZ400a master mix and the cells from well #12 to well #1 (instead of well #1 to well #12).

39. Place the 96-well plate in a plate reader equilibrated at 28°C and start measurement.
  ***Note:*** Measurements can also be done a physiologic temperature (i.e., 37°C) with similar results but of somewhat reduced quality due to faster turnover of the luciferase substrate and modest reductions in BRET response amplitudes. The choice of 28°C is motivated by the desire to maintain temperature constant across all experiments regardless of laboratory environmental conditions. Typically, the lowest temperature inside the instrument is 3°C–4°C above room temperature, so setting it up to 28°C ensures consistency regardless of range of temperature typically observed in the laboratory.
  a. Measure with same filters and integration settings as the kinetic measurements (see steps 26–27).
  b. Measure each minute for a total of 5 min.
    ***Note:*** Measurements will be done at six different time points (0, 1, 2, 3, 4, and 5 min) which covers times at which stimulation of most GPCRs will reach maximal response. See note below about processing of data corresponding to times.
40. To process the BRET data, export luminescence values from both the 535 nm and 450 nm channels.
  a. Divide luminescence counts in the acceptor channel by luminescence counts in the donor channel for each time point (BRET ratio) (Figure 3B).
  b. For all conditions, subtract the BRET ratio value of the well with no agonist (i.e., well 1) to get the difference in BRET relative to the buffer-only condition (ΔBRET [no agonist]) and multiplied by 1000 (ΔBRET·10^3^ [no agonist], Figure 3C) to get integer values which are more intuitive.

***Note:*** At this point, there should be 12 points, going from a value of 0 (corresponding to well 1, “no agonist”), that gradually increase until reaching a plateau at the highest concentrations of agonist.

***Note:*** Inspecting the data at each time point allows to determine whether responses have stabilized in time (i.e., BRET ratio values remain constant over consecutive times). In our hands, responses stabilize at 3–5 min for most GPCR-G protein combinations tested (∼100). It can also be useful to average two sequential time points to gain confidence in the accuracy of the measurement. For example, averaging the data from minutes 4 and 5 may result in a smoother curve and better representation of the dose dependent response.

41. Generate a three-parameter sigmoidal curve, assuming a Hill slope of 1, using an analysis software (e.g., GraphPad Prism).
  a. Pharmacological parameters like E_Max_ and EC_50_ can be extracted from this linear fit (Figure 3D).

***Note:*** This protocol can be scaled-up to measure activation of multiple GPCRs at the same time by using multiple rows of the 96-well plate.

***Note:*** In our hands, technical replicates are not required because the results are highly reproducible. However, it would be desirable to run technical replicates if the reproducibility becomes lower due to, for example, the use of an instrument with lower capabilities and/or the measurement of weak responses for a given GPCR-G protein combination.

## Expected outcomes

The protocols described here enable the measurement of G protein activity in live cells using ONE-GO biosensors in two assay formats. One, the kinetic assay (Figure 2), allows to do measurements with subsecond resolution in a lower throughput format, whereas the other, the endpoint assay (Figure 3), allows to do measurements with higher throughput but little time resolution.

With the data extracted from the kinetic assay, users will be able to generate traces that illustrate α2_A_-AR responses following agonist stimulation, which diminish upon antagonist treatment. Agonist stimulation by injection will rapidly trigger the formation of YFP-fused Gαi1-GTP, which will in turn interact with the Nluc-fused detector module and therefore increase BRET. Inversely, injection of the antagonist will reduce the amount of Gαi1-GTP, which will reduce BRET (Figure 2C). The change in BRET relative to the basal, unstimulated signal trace (i.e., ΔBRET (baseline)) represents the agonist-dependent GPCR response, showing the extent to which the GPCR changes the activation status the G protein (Figure 2D). For each different ONE-GO biosensor corresponding to a specific G protein and detector module combination, basal BRET values (i.e., prior to agonist stimulation) may differ. It can be useful to leverage publicly available information on the G protein coupling specific of any give GPCR of interest to decide on the ONE-GO biosensor to use. For example, the IUPHAR/BPS Guide to Pharmacology^24^ lists primary coupling preferences of GPCRs, and based on this annotation we have been able to detect responses for >70 GPCRs using ONE-GO biosensors.^1^ ONE-GO biosensors are also compatible with measuring responses elicited by endogenously expressed GPCRs,^1^ which is covered in a companion STAR protocol, although the expectation is that the magnitude of the responses will be significantly smaller. Likewise, the difference in BRET observed upon stimulation will not only depend on the strength of the receptor mediated response, but also on the intrinsic properties of the biosensors. This is because the affinity and relative orientation of G protein and detector module pairs are different for each biosensor. Monitoring basal BRET values and change in BRET upon stimulation of a well characterized receptor-G protein biosensor pair is valuable to assess internal consistency across experiments in a particular laboratory setting (i.e., instrument model, hardware specifications, cell transfections, etc.).

With the data extracted from the endpoint assay, users will be able to generate concentration-dependence curves and associated pharmacological parameters. Here, agonist effects are tested at a fixed time point with different concentrations of ligand. As concentrations of the agonist increase, more Gαi1-GTP is formed, thereby increasing BRET between the donor and acceptor in a concentration-dependent fashion (Figure 3B). It is important to note that while most GPCRs will reach maximum activation and stabilize after 3–5 min, this should be assessed on a case-by-case basis to adjust data processing protocols (i.e., choose the time point(s) that represent activation best). The endpoint method allows for the testing of multiple conditions simultaneously (e.g., testing different agonist concentrations), though it sacrifices the temporal resolution afforded by the kinetic assay. In this assay, the change in BRET relative to the basal, unstimulated signal (i.e., ΔBRET (no agonist)) represents the agonist-dependent GPCR response, showing the extent to which the GPCR stimulates the G protein (Figure 3C). From these data points, users can plot a three-parameter sigmoidal curve (or another curve fit they deem adequate for their experiments), allowing for the determination of the E_Max_ and/or the EC_50_. The E_Max_ represents the maximal activation of the GPCR/G protein combination under study, which reflects efficacy, while the EC_50_ represents the concentration of agonist required to achieve 50% of the maximal response, which reflects potency (Figure 3D). While the example provided here focuses on one GPCR (α2_A_-AR), this protocol can easily be scaled up to test numerous GPCRs in parallel by using one row of the 96-well plate for each GPCR. For example, scaling up the protocol described here to testing eight different 12-point concentration curves simultaneously in one 96-well plate can easily be achieved with a multi-channel pipet. The design of ONE-GO biosensors as a single plasmid design is also conducive to higher throughput assay formats by reducing the workflow bottleneck imposed by more complex biosensor systems which require the transfection of three to four plasmids instead of one. A comparison of different biosensor systems for detecting GPCR responses has been covered in detail in a recent review.^25^

## Limitations

While this protocol describes how to detect activity of the α2_A_-AR using the Gαi1 ONE-GO, detection of responses elicited by other receptors might not be detectable or equally robust. While we have successfully measured responses triggered by > 70 GPCRs,^1^ a few receptors tested did not yield positive responses. Similarly, ONE-GO biosensors for different types of G proteins lead to responses of different BRET amplitude, which can limit sensitivity and/or reproducibility of results for those in which the dynamic range is smaller. Similarly, there are so far no ONE-GO biosensors for G_olf_, G_t_, G_gust_, G_11_, G_14_ and G_15_, precluding the study of responses by these particular G proteins using the protocols described here. From a technical standpoint, although many plate readers that allow for luminescence measurements should be compatible with this protocol, instruments with more sensitive detectors, and, especially, instruments capable of simultaneous dual detection of donor and acceptor channels are preferred. This is because these features reduce detection noise, thereby improving the confidence of measurements, which is particularly critical when differences in BRET are small. Similarly, the use of plate readers that allow to record while injecting, as opposed to those that require to stop recording during injections, allows for better kinetic measurements without loss of information.

## Troubleshooting

### Problem 1

Weak luminescence signal of biosensor components.

### Potential solution

Low expression of the biosensor components is the likely cause of the weak luminescence signal detection. This can be due to a poor transfection efficiency. Making sure that all transfection reagents (e.g., plasmids, 2x HBS, 2M CaCl_2_) have been made correctly and/or have not degenerated with time can be a solution (see step **#13**). One critical aspect is the adjustment of pH for the 2x HBS solution: pH should be brought up precisely to 7.4, while never going above that point throughout the process (discard the solution if pH goes above 7.4 at any point, do not try to readjust). Another potential cause of low signal is poor cell health at the time of transfection. It is important to ensure that cell media is made correctly and cells are not kept at either too low or high confluency levels (see step **#1**). If the luminescence signal is weak but BRET responses are still detectable, increasing the integration time can help reduce the noise of the recording, at the expense of time resolution (see step **#26**). Once optimal BRET assay conditions have been established with a particular method of transfection, even if different from the one described here, like PEI or lipofectamine, we recommend continuing with that method. It is important to be aware that the specific amounts of plasmid to be transfected to obtain optimal results will depend on many factors, including method of transfection, cell status, or quality of the DNA preparations.

### Problem 2

Marked increases or decreases of BRET ratio prior to agonist injection (“basal slopes”).

### Potential solution

The baseline BRET signal might not be straight due to either too high or too low expression of the biosensor components. When setting experimental conditions, it is advisable to do a titration of the amount of ONE-GO biosensor plasmid transfected to establish optimal amounts (see step **#11**). Another potential cause of marked slopes in the baseline BRET signal is that the quality of luciferase substrate has deteriorated with time. This can be addressed by taking a fresh aliquot of the luciferase substrate. In some cases, when none of the strategies proposed above has fixed the issue, we have had success by changing the concentration of substrate used and/or changing the time of incubation before starting the measurement (see step **#26**).

### Problem 3

Luminescence counts are high, but no BRET response is detected upon agonist stimulation.

### Potential solution

One potential explanation is that the cognate receptor is not expressed, which could be addressed by repeating the experiment transfecting a new DNA preparation of the plasmid of interest and by performing controls using a receptor known to activate the G protein of interest. A less intuitive reason for this problem is that the expression of the biosensor components is too *high*. While it may seem logical to believe that more biosensor expression is better, the opposite is actually true. High expression of the sensor can lead to an artificially high basal BRET ratio which can mask any responses. Ideally, expression of ONE-GO biosensors (and many other BRET-based biosensors) should be as low as possible while allowing to obtain reliable luminescence measurements. This is conducible to BRET responses with a larger dynamic range accompanied by low noise (see step **#11**).

### Problem 4

Luminescence counts are high and GPCR plasmids DNA is adequate, but no BRET response is detected upon agonist stimulation.

### Potential solution

Some receptors are also susceptible to getting desensitized in culture medium. Starving cells in media containing reduced amounts of serum (e.g., 0.1% FCS instead of 10%) can sometimes help keeping the receptor at the plasma membrane (see step **#17**). Finally, it is important to transfect the correct combination of GPCR/ONE-GO sensor (see step **#11**). GPCRs may have more or less selectivity towards which G proteins they can couple to and activate. It may be useful to check the literature for specific GPCR/G protein coupling, or databases such as the IUPHAR/BPS Guide to Pharmacology which lists primary G protein coupling for each receptor.

### Problem 5

Luminescence counts are high and GPCR expression is adequate, but no BRET response is detected upon agonist stimulation.

### Potential solution

This scenario may be due to a deterioration in the agonist used. It is often helpful to prepare new agonist dilutions with fresh compounds to address this issue. In general, it is important to avoid freeze-thaw cycles, for example by making aliquots of chemicals in use (see step **#27**).

## Resource availability

### Lead contact

Further information and requests for resources and reagents should be directed to and will be fulfilled by the lead contact, Mikel Garcia-Marcos (mgm1@bu.edu).

### Technical contact

Technical questions on executing this protocol should be directed to and will be answered by the technical contact, Remi Janicot (rjanicot@bu.edu).

### Materials availability

All ONE-GO biosensor plasmids and associated construct are available via Addgene (ONE-GO biosensors kit, #1000000224).

### Data and code availability

The associated published article^1^ includes all datasets generated or analyzed in this study.

## Acknowledgments

This work was primarily supported by a grant from the National Institutes of Health (R01GM147931 to M.G.-M.). R.J. is supported by a Predoctoral Fellowship from the American Heart Association (898932).

## Author contributions

R.J. and M.G.-M. wrote the manuscript. M.G.-M. supervised the project and acquired funding.

## Declaration of interests

The authors declare no competing interests.
